# IM156, a new AMPK activator, protects against polymicrobial sepsis

**DOI:** 10.1111/jcmm.17341

**Published:** 2022-05-02

**Authors:** Ji Hyeon Kang, Sung Kyun Lee, Nam Joo Yun, Ye Seon Kim, Jason Jungsik Song, Yoe‐Sik Bae

**Affiliations:** ^1^ Department of Biological Sciences Sungkyunkwan University Suwon Republic of Korea; ^2^ 65680 Center for Convergent Research of Emerging Virus Infection Korea Research Institute of Chemical Technology Daejeon Republic of Korea; ^3^ 37991 Division of Rheumatology Department of Internal Medicine Yonsei University College of Medicine Seoul Republic of Korea; ^4^ 37991 Institute for Immunology and Immunological Diseases Yonsei University College of Medicine Seoul Republic of Korea

**Keywords:** AMP‐activated protein kinase, caecal ligation and puncture, IM156, neutrophil extracellular traps, sepsis

## Abstract

IM156, a novel biguanide with higher potency of AMP‐activated protein kinase activation than metformin, has inhibitory activity against angiogenesis and cancer. In this study, we investigated effects of IM156 against polymicrobial sepsis. Administration of IM156 significantly increased survival rate against caecal ligation and puncture (CLP)‐induced sepsis. Mechanistically, IM156 markedly reduced viable bacterial burden in the peritoneal fluid and peripheral blood and attenuated organ damage in a CLP‐induced sepsis model. IM156 also inhibited the apoptosis of splenocytes and the production of inflammatory cytokines including IL‐1β, IL‐6 and IL‐10 in CLP mice. Moreover, IM156 strongly inhibited the generation of reactive oxygen species and subsequent formation of neutrophil extracellular traps in response to lipopolysaccharide in neutrophils. Taken together, these results show that IM156 can inhibit inflammatory response and protect against polymicrobial sepsis, suggesting that IM156 might be a new treatment for sepsis.

## 1 INTRODUCTION

Sepsis is an intractable emergency infectious disease with high mortality and morbidity.^1^ Despite improved healthcare during the last several decades, currently approximately 19.4 million cases of severe sepsis occur annually in the world.^1^ Since there is no effective therapeutic agent to control sepsis, developing treatments for sepsis has been an important issue.

Neutrophils play crucial roles in innate defence activity against invading pathogens.^2^ They can remove pathogenic microorganisms via phagocytosis, generation of reactive oxygen species and degranulation.^3^ Neutrophil extracellular trap (NET) is a specialized lattice structure containing DNA, antimicrobial peptides and myeloperoxidase. It contributes to defence activity of neutrophils.^4^ However, increased NET formation has been implicated as a mechanism to induce adverse effects in several inflammatory diseases, resulting in increased mortality.^5^, ^6^ In sepsis, although NET can be used to effectively remove invading pathogens, NET can induce tissue damage in the host.^7^, ^8^ Therefore, developing molecules that can control NET formation to treat sepsis is essentially needed.

IM156, formerly known as HL156, is a novel biguanide with more potency than metformin and phenformin in terms of AMP‐activated protein kinase (AMPK) activation.^9^ AMPK is an essential enzyme that regulates energy metabolism to prevent cell damage. It plays a central role in various inflammatory responses.^10^ Since AMPK activation can induce beneficial outcomes in pathological progression of diverse human diseases,^11^, ^12^, ^13^ some AMPK activators such as metformin have been proposed as new therapeutic agents to control different types of disorders such as type 2 diabetes.^14^ Previously, it has been reported that IM156 can inhibit angiogenesis in gastric cancer cells^9^ and reverse multidrug resistance in human multidrug‐resistant cancer cells.^15^ Mechanistically, IM156 inhibits mitochondrial respiration in cancer cells.^9^ It also shows protective activity against peritoneal fibrosis in mice.^16^, ^17^ However, roles of IM156 in the pathogenesis of sepsis and neutrophil activity have not been reported yet.

Thus, the objective of this study was to determine whether IM156 might possess therapeutic effects against polymicrobial sepsis, an experimental animal model of sepsis. The mode of action involved in the anti‐septic effects of IM156 was investigated in an animal model. We also examined *in vitro* effects of IM156 on neutrophil activity by focusing on NET formation.

## 2 MATERIALS AND METHODS

### 2.1 Animals and caecal ligation and puncture (CLP) experimental sepsis model

All animal experiments were approved by the Institutional Review Committee for Animal Care and Use at Sungkyunkwan University (Suwon, Korea). Male wild‐type ICR mice (eight‐week‐old) were purchased from Orient Bio (Seongnam, Korea). Polymicrobial sepsis was induced by CLP as described previously.^18^ In brief, mice were anaesthetized with isoflurane. The caecum was exposed from an abdominal midline incision. The caecum was then ligated below the ileocaecal valve and punctured twice through both surfaces (or once for cytokine levels measurement) using a 22‐gauge needle, and the abdomen was closed. Sham‐operated mice were subjected to the same procedure without puncture or ligation of the caecum. IM156 was kindly provided by ImmunoMet Therapeutics (Houston, TX, USA). Vehicle (distilled water (DW)) or IM156 (5, 10 and 15 mg/kg) was injected subcutaneously. Survival was monitored daily for 10 days.

### 2.2 Tissue histology

Mice were sacrificed at 24 h after surgery. Their lungs and spleen were isolated for paraffin sectioning. Tissues were fixed in 10% (v/v) neutral‐buffered formalin (NBF) for 1 day at 37°C and then embedded with paraffin. Tissue blocks were cut into 4‐μm‐thick slices and stained with haematoxylin and eosin for histological analysis.^19^ For terminal deoxynucleotidyl transferase dUTP nick end labelling (TUNEL) assay, paraffin‐embedded tissue sections were permeabilized with Triton X‐100 at 4°C for 2 min and flooded with the in‐situ Cell Death Detection Kit (Roche, Basel, Switzerland) for 60 min at 37°C. The percentage of TUNEL‐positive cells from splenocytes was quantified under a light microscope as described previously.^20^, ^21^

### 2.3 Measurement of bactericidal activity *in vivo*

To determine the bactericidal activity of IM156 in the peritoneum and blood, peritoneal lavage fluid and peripheral blood were collected at 24 h after CLP. Each sample was diluted with sterile phosphate‐buffered saline (PBS) and incubated overnight on blood‐agar base plates (Trypticase Soy Agar Deeps; Becton Dickinson, Franklin Lakes, NJ, USA) at 37°C. Colony‐forming units (CFUs) were counted as described previously.^18^

### 2.4 Measurement of cytokine production

To measure levels of cytokine induced by polymicrobial sepsis, peritoneal lavage fluid was collected at 24 h after CLP. After removing cells in fluid through centrifugation at 8000 rpm for 5 min, cytokine levels were measured with an enzyme‐linked immunosorbent assay (ELISA; eBioscience Inc., San Diego, CA, USA) using antibody pairs according to the manufacturer's instruction.

### 2.5 Isolation of neutrophils

Bone marrow cells of wild‐type ICR were isolated from femurs and tibias with sterile PBS. The cell suspension was centrifuged at 1500 rpm for 5 min. Next, resuspended cells were carefully loaded on 45% and 60% Percoll (Cytiva, Marlborough, MA, USA) gradient and centrifuged at 2580 rpm for 25 min with low breaking. Cells were isolated on the 45–60% interface layer, and RBCs were removed by hypotonic lysis. Isolated cells were >95% Ly6G‐positive by FACS Canto Ⅱ (BD Biosciences, San Jose, CA, USA).

### 2.6 Measurement of NET formation by fluorescence microscopy

Bone marrow‐derived neutrophils (2 × 10^5^ cells) were seeded to 0.01% poly‐_L_‐lysine‐coated 24‐well plates and stimulated with lipopolysaccharide (LPS) (50 μg/ml) (Sigma, St Louis, MO, USA) for 4 h in the presence of vehicle (DW) or IM156 pre‐incubation. At the end of stimulation, cells were fixed with 4% paraformaldehyde and then stained with SYTOX Green nucleic acid stain (5 μM) for 15 min. Stained NETs were visualized with an OPTINITY Inverted Microscope (Korea Lab Tech, Seongnam, Korea). Images were taken using a Nikon digital camera. NETs (SYTOX‐positive) area was quantified using NIH ImageJ software.

### 2.7 Western blot analysis

Bone marrow‐derived neutrophils (7×10^5^ cells) were stimulated with ionomycin (5 μM) for 2 h in the presence of vehicle (DW) or IM156. After stimulation, cells were collected and lysed in radioimmunoprecipitation assay buffer (RIPA buffer) supplemented with protease inhibitor cocktails (Sigma, St Louis, MO, USA) on ice. Extracted proteins were separated by sodium dodecyl sulphate‐polyacrylamide gel electrophoresis (SDS‐PAGE) (15% gel) and transferred to a polyvinylidene difluoride membrane (Millipore, Billerica, MA, USA). Levels of citrullinated histone 3 were detected using an anti‐histone H3 antibody (citrulline R2 + R8 + R17, Abcam, Cambridge, MA, USA) as described previously.^7^ To confirm equal loading, β actin was detected using an anti‐β‐actin antibody (Abcam). Relative Cit‐H3 expression levels were normalized to the β‐actin expression level.

### 2.8 Measurement of reactive oxygen species (ROS)

ROS were measured by flow cytometry with H_2_‐DCF‐DA (Sigma, St Louis, MO, USA) stain. Bone marrow‐derived neutrophils (5 × 10^5^ cells) were stimulated with LPS (50 μg/ml) for 30 min in the presence of vehicle (DW) or IM156 pre‐incubation and then loaded with 10 μM DCF‐DA for 30 min at 37°C. Stained cells were washed, resuspended and immediately placed on ice. Fluorescence was measured by flow cytometry (FACSCanto Ⅱ) and analysed using FlowJo analytical software (BD Biosciences, San Jose, CA, USA).

### 2.9 Quantitative RT‐PCR (qRT‐PCR)

Total RNA was isolated from LPS‐stimulated neutrophils treated with or without IM156 using TRIzol reagent (Life Technology, Carlsbad, CA, USA) according to the manufacturer's protocol. Isolated RNA was used to synthesize cDNA using a Maxime RT PreMix Kit (Intron, Seongnam, Korea) Quantitative polymerase chain reaction (qPCR) was performed with a Rotor‐Gene SYBR Green PCR Kit (QIAGEN, Hilden, Germany) and gene‐specific primers: *Nrf2*‐forward, 5'‐TTGGCAGAGACATTCCCAT‐3'; *Nrf2*‐reverse, 5'‐GCTGCCACCGTC‐ACTGGG‐3'; *Hmox1*‐forward, CAGCCCCACCAAGTTCAAAC‐3'; *Hmox1*‐reverse, 5'‐GGCGGTCTTAGCCTCTTCTGT‐3'; *Nqo1*‐forward, 5'‐GGTTTACAGCATTGGCCACAC‐T‐3'; *Nqo1*‐reverse, 5'‐AACAGGCTGCTTGGAGCAAA‐3'; *Gapdh*‐forward, 5'‐TCCACCA‐CCCTGTTGCTGTA‐3'; and *Gapdh*‐reverse, 5'‐AATGTGTCCGTCGTGGAT‐CT‐3'. For qPCR, 55 PCR cycles were performed in three steps including denaturation (95°C, 30 s), annealing (60°C, 30 s) and extension (72°C, 1 min). Relative gene expression levels were normalized to the *Gapdh* expression level.^19^, ^22^

### 2.10 Statistical analysis

All results were evaluated via GraphPad Prism software. They are expressed as the mean ± standard error of the mean (SEM). The statistical analysis was performed using Student's *t*‐test or analysis of variance (ANOVA). Survival data were analysed using the log‐rank test. *p* ≤ 0.05 was considered statistically significant.

## 3 RESULTS

### 3.1 IM156 protects against CLP‐induced mortality

**FIGURE 1:**
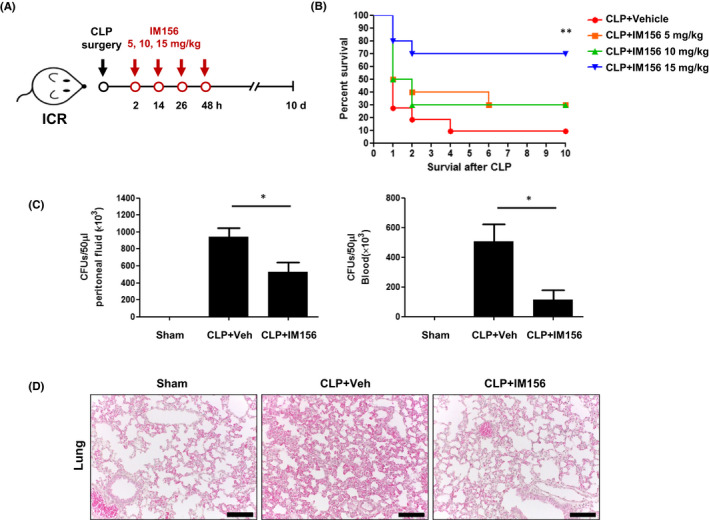
Therapeutic effects of IM156 against caecal ligation and puncture (CLP)‐induced sepsis. (A) Schematic illustration of the *in vivo* experiment corresponding to (B). (B) Different doses (0, 5, 10 and 15 mg/kg) of IM156 were injected subcutaneously into CLP mice at 2, 14, 26 and 38 h post‐CLP. Survival rates were monitored for 10 days. **p < 0.01 compared to vehicle control by analysis of variance (ANOVA). Sample size: *n* = 10–11 mice/group. (C, D) IM156 (15 mg/kg) was injected subcutaneously into CLP mice at 2 and 14 h post‐CLP. Mice were sacrificed 24 h after CLP surgery. (C) Colony‐forming units (CFUs) were determined from blood or peritoneal lavage fluid. (D) Lungs were stained with haematoxylin and eosin (magnification, ×100). Data are expressed as the mean ± standard error of the mean (SEM) (*n* = 8 for C). *p < 0.05 by Student's *t*‐test. Scale bar, 100 μm (D)

We first examined whether IM156 had therapeutic effects against polymicrobial sepsis in the CLP model. As shown in Figure 1A, administration of IM156 four times at 12‐h intervals from 2 h after CLP establishment elicited therapeutic effects against sepsis. Notably, 15 mg/kg of IM156 significantly improved the survival rate by 70% compared to just 10% of the CLP control group (Figure 1B).

Next, we investigated the mode of action in the beneficial effects of IM156 in the sepsis model. In the early stages of sepsis, failure to remove bacteria from the infected sites can lead to systemic inflammation in the blood. Due to the leakage of viable bacteria from the caecum to the lumen during CLP surgery, CLP‐induced mortality is strongly associated with bacterial colony counts in peripheral blood and peritoneal fluid.^23^ Based on the improved survival rate with IM156 administration (Figure 1B), we examined bactericidal effects of IM156 in the CLP model. Bacterial colony counts were increased after CLP surgery. However, they were markedly decreased (by ~40% in the peritoneal lavage fluid and by ~80% in the peripheral blood) after IM156 administration (Figure 1C). Sepsis is a typical respiratory disease caused by infectious bacteria that can lead to severe lung inflammation.^23^ In this study, we also observed that CLP caused lung damage with alveolar wall congestion, formation of thrombotic lesions and abundant infiltration of inflammatory cells (Figure 1D). Lung histopathology was significantly resolved after administration of IM156, leading to improved survival (Figure 1D). These results suggest that IM156 can effectively decrease mortality by increasing bactericidal activity and attenuating lung damage in the CLP‐induced sepsis model.

### 3.2 IM156 inhibits CLP‐induced splenocyte apoptosis and inflammatory cytokine production *in vivo*

**FIGURE 2:**
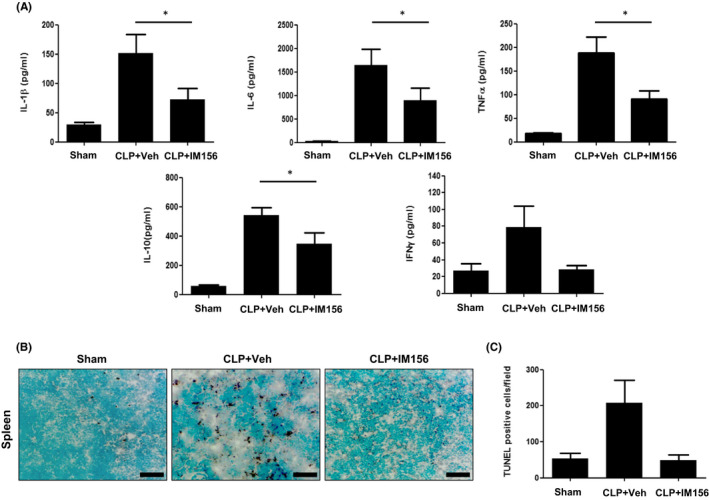
Inhibitory effects of IM156 on caecal ligation and puncture (CLP)‐induced inflammatory cytokine production and leucocyte apoptosis. (A‐C) Vehicle (distilled water (DW)) or IM156 (15 mg/kg) was injected into CLP mice at 2 and 14 h post‐CLP. (A) Peritoneal fluids were collected at 24 h after CLP. Cytokine levels in the peritoneal fluid were determined by enzyme‐linked immunosorbent assay (ELISA). (B) Spleens were collected from mice in sham, CLP plus vehicle or CLP plus IM156 administration group at 24 h after CLP and used in a terminal deoxynucleotidyl transferase dUTP nick end labelling (TUNEL) assay. (C) TUNEL‐positive cells from spleens of mice described in (B) were quantified. Data are expressed as the mean ± standard error of the mean (SEM) (*n* = 8 for A and *n* = 6–10 for C). *p < 0.05 by Student's *t*‐test. Scale bar, 100 μm (B)

Sepsis is a systemic inflammatory response induced by infection.^1^ During the pathological progress of sepsis, several inflammatory cytokines such as IL‐1β, IL‐6, TNF‐α and IFN‐γ are excessively produced.^24^ Therefore, it is important to reduce levels of these proinflammatory cytokine to control sepsis. In this study, we also found that CLP surgery markedly increased levels of IL‐1β, IL‐6, TNF‐α and IFN‐γ in the peritoneal fluid. However, administration of IM156 significantly decreased their production in the animal model (Figure 2A). IM156 also slightly reduced IL‐10 levels in peritoneal fluid of mice (Figure 2A).

Previous studies have suggested that immune cell apoptosis including CD4 T cells, B cells and dendritic cells is a possible cause of progressive organ failure in sepsis.^25^ As shown in Figure 2B, CLP‐induced sepsis increased apoptosis of lymphocytes in spleen as detected by TUNEL histology. IM156 administration significantly attenuated apoptosis of splenocytes and decreased the number of apoptotic cells compared to the vehicle group (Figure 2B‐C). These results suggest that IM156‐induced anti‐septic activity could be mediated by inhibition of inflammatory cytokine production and apoptosis of lymphocytes in the CLP model.

### 3.3 IM156 strongly blocks NET formation in mouse neutrophils

**FIGURE 3:**
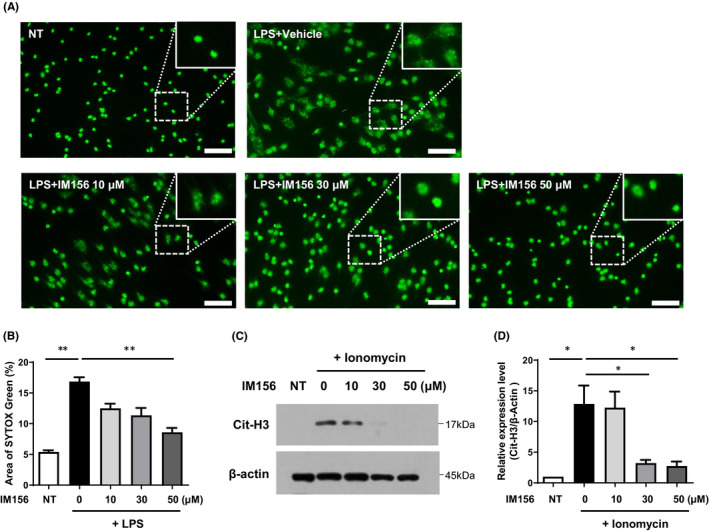
Inhibitory effects of IM156 on neutrophil extracellular trap (NET) formation *in vitro*. (A‐D) Neutrophils isolated from ICR mice were stimulated with lipopolysaccharide (LPS) (50 μg/ml, A‐B) or ionomycin (5 μM, C‐D) for 4 h in the presence of vehicle (distilled water (DW)) or IM156 (10, 30 and 50 μM) pre‐incubation. (A) NET formation was measured using SYTOX Green Nucleic acid staining and visualized by fluorescence microscopy. Insertion represents enlargement of the square area. Scale bar, 50 μm. (B) Area of SYTOX Green per field described in (A) was quantified. (C) Citrullinated histone 3 (Cit‐H3) levels were measured by Western blot analysis. (D) Relative expression levels of citrullinated‐histone 3/β‐actin were quantified. Data are expressed as the mean ± standard error of the mean (SEM). *p < 0.05 and **p < 0.01 by Student's *t*‐test. Scale bar, 50 μm (A). NT, no treatment

Considering that NET formation is one of the important mechanisms involved in the pathogenesis of some respiratory disorders including sepsis and COVID‐19 infection,^26^ we investigated whether IM156 could affect NET formation in mouse neutrophils. As shown in Figure 3A, stimulation of bone marrow neutrophils isolated from ICR mice with LPS (50 μg/ml) for 4 hours induced strong NET formation, which was measured by fluorescence microscopy with a cell‐impermeable dye and SYTOX Green nucleic acid stain. Treatment with IM156 at several concentrations prior to LPS stimulation for 30 min attenuated NET formation in response to LPS (Figure 3A). At 10 μM, inhibitory effects of IM156 on LPS‐induced NET formation were apparent. IM156 at 50 μM significantly blocked LPS‐induced NET formation (Figure 3B). These results indicate that IM156 can effectively inhibit NADPH‐oxidase (NOX)‐dependent NET formation in response to LPS.

Unlike NOX‐dependent NET formation by LPS, NOX‐independent NET formation is accompanied by histone 3 citrullination in neutrophils.^27^ Here, we found that IM156 strongly inhibited histone 3 citrullination in response to ionomycin (5 μM). Western blot analysis revealed that addition of 30–50 μM IM156 almost completely blocked ionomycin‐induced histone 3 citrullination (Figure 3C‐D). These results suggest that IM156 may also block NOX‐independent NET formation by inhibiting histone 3 citrullination.

### 3.4 IM156 inhibits ROS generation via upregulation of antioxidant genes expression

**FIGURE 4:**
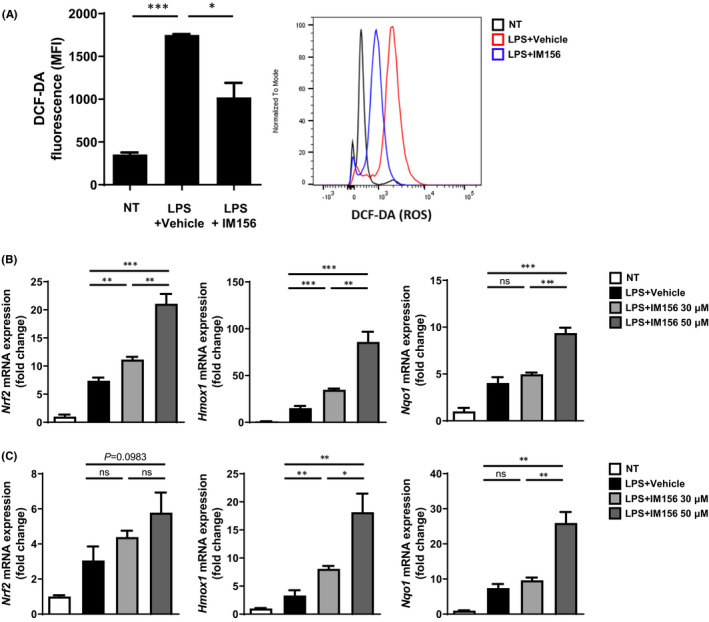
IM156 inhibits reactive oxygen species (ROS) generation in neutrophils. (A) Neutrophils isolated from ICR mice were stimulated with lipopolysaccharide (LPS) (50 μg/ml) for 1 h in the presence of vehicle (distilled water (DW)) or IM156 (50 μM) pre‐incubation for 30 min. Levels of ROS were measured using flow cytometry with H_2_‐DCF‐DA stain. (B‐C) Neutrophils were stimulated with LPS (50 μg/ml, B or 100 ng/ml, C) for 6 h in the presence of vehicle (DW) or IM156 (30 and 50 μM) pre‐incubation. Quantitative real‐time polymerase chain reaction (qPCR) was used to measure mRNA expression levels of antioxidant genes (*Nrf2*, *Hmox1* and *Nqo1*). Data are expressed as the mean ± standard error of the mean (SEM). *p < 0.05 and ***p < 0.001 by Student's *t*‐test

Previously, ROS generation has been regarded as an important prerequisite for the efficient NET formation.^27^ Since IM156 strongly inhibited NET formation in mouse neutrophils (Figure 3), we then examined whether IM156 could affect ROS generation in neutrophils. Stimulation of mouse bone marrow neutrophils with LPS (50 μg/ml) for 1 h strongly increased ROS generation (Figure 4A). The addition of IM156 (50 μM) prior to LPS stimulation significantly inhibited ROS generation in mouse neutrophils (Figure 4A). These results suggest that IM156 might negatively regulate cellular responses that are ROS‐dependent in neutrophils.

Since IM156 strongly inhibited ROS generation in response to LPS in mouse neutrophils, we next examined effects of IM156 on the expression of several antioxidant genes. At first, we observed that stimulation of mouse neutrophils with LPS (50 μg/ml and 100 ng/ml) for 6 h strongly increased expression levels of antioxidant genes such as *Nrf2*, *Hmox1* and *Nqo1* in neutrophils (Figure 4B‐C). The addition of IM156 prior to stimulation of mouse neutrophils with LPS (50 μg/ml) enhanced the expression of these antioxidant genes (Figure 4B). IM156 also markedly increased the expression of antioxidant genes when mouse neutrophils were stimulated with a low concentration (100 ng/ml) of LPS (Figure 4C). Collectively, these results suggest that IM56 can upregulate antioxidant genes to decrease ROS generation in mouse neutrophils in response to LPS, which can lead to attenuation of NET formation.

## 4 DISCUSSION

Sepsis, a representative infectious disease, is a pathological condition that kills more than 11 million people annually.^28^ Although severe sepsis and septic shock have increased significantly over the past 20 years, currently there is no way to treat sepsis without antibiotics.^24^ Thus, development of effective therapeutic agents against severe sepsis is urgently needed. In this study, we demonstrate that administration of IM156, a new metformin derivative, can elicit strong therapeutic effects against polymicrobial sepsis in an animal model. Our results suggest that IM156 can be considered as a useful material to develop anti‐septic drugs.

Under normal cellular and physiological conditions, cells maintain metabolic homeostasis through a highly coordinated system. AMPK is one of the most important metabolic regulators that play dual roles in energy consumption and accumulation.^12^, ^29^ Functionally, activation of AMPK can promote ATP production through mitochondrial biogenesis and glycolysis in the stress condition. In contrast, the AMPK pathway limits ATP‐consuming processes such as fatty acid and cholesterol synthesis.^30^ Recent studies have shown that AMPK activation by drugs and compounds is effective in treating experimental septic mice,^12^, ^31^, ^32^ suggesting that AMPK is an important target to modulate sepsis pathogenesis. In this study, we demonstrated that administration of IM156, a novel biguanide with more potent than metformin, improved survival rate and blocked lung damage in a well‐established experimental sepsis model. IM156 showed therapeutic effects against polymicrobial sepsis through several mechanisms: (1) causing bactericidal activity, (2) inhibiting apoptosis of lymphocytes and (3) reducing the production of inflammatory cytokines. Our results support the notion that AMPK activation is a good approach to treat sepsis. Moreover, IM156, a very potent AMPK activator, might be a candidate for an effective drug to control sepsis.

CLP‐induced sepsis animal model features two distinct phases.^24^ The early stage of sepsis is considered a hyperinflammatory phase that is mediated primarily by macrophages, neutrophils and monocytes stimulated by microorganisms. The hallmark of this phase is increased production of inflammatory cytokines, usually called a ‘cytokine storm’. Cytokines such as IL‐1β, IL‐6 and TNF‐α are important in eliminating infection. However, excessive production can cause tissue and organ damage.^33^ Here, we also observed that increased cytokine production after CLP surgery. Such increase was markedly inhibited by IM156 administration (Figure 2A). These results suggest that IM156 can induce the attenuation of lung damage by reducing cytokine levels. The late phase (>12 h following the onset of sepsis) includes loss of phagocytic function and increased apoptotic death of immune effector cells, particularly dendritic cells and lymphocytes, which can lead to an immune‐suppressive condition.^24^, ^34^ It is well known that the loss of functional lymphocytes caused by apoptosis directly contributes to organ failure, susceptibility to the second infection and overall septic mortality.^35^ In addition, the degree of apoptosis of lymphocytes is useful for determining the severity of sepsis.^24^ IM156 significantly enhanced the anti‐apoptotic effect on lymphocytes in CLP‐induced sepsis (Figure 2B‐C). Therefore, IM156 can prevent sepsis‐induced immunosuppression by inhibiting apoptosis of lymphocytes, ultimately improving the survival rate of CLP mice.

Meanwhile, neutrophils with roles in innate defence activity against sepsis are rapidly increased due to granulopoiesis and delayed apoptosis within 24 h after sepsis initiation.^24^ Excessive production of immature neutrophils can cause persistent dysfunction such as abnormal cytokine production and oxidative burst which can lead to a paralysed immune response in sepsis.^3^ One of the main functions of neutrophils is NET formation that can remove circulating bacteria in the bloodstream and provide intravascular immunity to septic infections.^7^ Although the beneficial role of NETs during sepsis has been demonstrated, some evidence indicates that NETs and their components can also increase complications of sepsis, such as thrombosis.^6^ A recent study has revealed that treatment with metformin, an AMPK activator, can reduce concentrations of NET components in the septic mice.^36^ This effect was related to the inhibitory effect exerted by metformin on PKC‐NADPH oxidase (NOX) pathway. By conducting in vitro experiments, we observed that IM156 inhibited LPS‐induced NET formation (Figure 3A‐B) and almost completely reduced ionomycin‐induced histone 3 citrullination (Figure 3C‐D). Since LPS‐induced NET formation initiates ROS production by NADPH oxidase activation, we additionally investigated antioxidant effects of IM156. Upon LPS stimulation, IM156 strongly blocked ROS production in mouse neutrophils (Figure 4A) and increased the expression of antioxidant genes such as *Nrf2*, *Hmox1* and *Nqo1* (Figure 4B‐C). Collectively, these results demonstrate that IM156 can upregulate antioxidant gene expression to block ROS production, leading to suppression of NET formation.

In conclusion, IM156, a potent AMPK activator, shows several protective effects against polymicrobial sepsis by modulating bacterial clearance, cytokine storm and apoptosis of lymphocytes. At the cellular level, we also observed the inhibitory effect of IM156 on NET formation in response to LPS. Mechanistically, IM156 can inhibit ROS generation by upregulating antioxidative effects, which can lead to reduced NET formation. Taken together, our results suggest that IM156 might be a potential candidate to develop therapeutic agents to control sepsis.

## AUTHOR CONTRIBUTION

**Ji Hyeon Kang:** Conceptualization (equal); Formal analysis (equal); Investigation (equal); Writing – original draft (equal); Writing – review & editing (equal). **Sung Kyun Lee:** Conceptualization (equal); Formal analysis (equal); Investigation (equal); Writing – original draft (equal); Writing – review & editing (supporting). **Nam Joo Yun:** Formal analysis (supporting); Investigation (supporting); Writing – review & editing (supporting). **Ye Seon Kim:** Formal analysis (supporting); Investigation (supporting); Writing – review & editing (supporting). **Jason Jungsik Song:** Conceptualization (equal); Funding acquisition (equal); Writing – original draft (equal); Writing – review & editing (equal). **Yoe‐Sik Bae:** Conceptualization (equal); Funding acquisition (equal); Supervision (equal); Writing – original draft (equal); Writing – review & editing (equal).

## CONFLICTS OF INTEREST

The authors have no conflicts of interest to disclose.

## Data Availability

The data that support the findings of this study are available from the corresponding author upon reasonable request.
